# Myeloid Cell Classification and Therapeutic Opportunities Within the Glioblastoma Tumor Microenvironment in the Single Cell-Omics Era

**DOI:** 10.3389/fimmu.2022.907605

**Published:** 2022-06-16

**Authors:** Collin J. Larkin, Víctor A. Arrieta, Hinda Najem, Gongbo Li, Peng Zhang, Jason Miska, Peiwen Chen, Charles David James, Adam M. Sonabend, Amy B. Heimberger

**Affiliations:** ^1^ Department of Neurological Surgery, Northwestern University Feinberg School of Medicine, Chicago, IL, United States; ^2^ Lou and Jean Malnati Brain Tumor Institute of the Lurie Comprehensive Cancer Center, Northwestern University Feinberg School of Medicine, Chicago, IL, United States; ^3^ Programa de Estudios Combinados en Medicina (PECEM), Facultad de Medicina, Universidad Nacional Autónoma de México, Mexico City, Mexico

**Keywords:** glioblastoma, immunotherapy, macrophage, tumor microenvironment, single cell analysis, transcriptomics, spatial analysis

## Abstract

The glioma tumor microenvironment (TME) is complex and heterogeneous, and multiple emerging and current technologies are being utilized for an improved comprehension and understanding of these tumors. Single cell analysis techniques such as single cell genomic and transcriptomic sequencing analysis are on the rise and play an important role in elucidating the glioma TME. These large datasets will prove useful for patient tumor characterization, including immune configuration that will ultimately influence therapeutic choices and especially immune therapies. In this review we discuss the advantages and drawbacks of these techniques while debating their role in the domain of glioma-infiltrating myeloid cells characterization and function.

## Introduction

Gliomas are composed of multiple distinct cell populations, each playing a unique role within the tumor microenvironment (TME). Each of these cell types contains a spectrum of subtypes that increase the level of heterogeneity and complexity of these tumors. In this context, antagonistic forces promoting both tumor growth and suppression exist in the TME that influence clinical outcomes and responses to therapies. Initiatives such as The Cancer Genome Atlas (TCGA) and the GLASS consortium (1), have provided important information about the genetic variation and evolution among gliomas, leading to the molecular classification for glioblastoma (GBM) (2). However, since bulk genomic and transcriptome data averages the genetic alterations and gene expression patterns, respectively, of individual tumors, the analysis of such data has limits regarding determining the extent of cell subpopulation heterogeneity within a tumor and thus, response to therapeutic interventions. In contrast, technologies such as single-cell RNA-seq (scRNA-seq) and cytometry by time-of-flight (CyTOF) are enabling the high-resolution characterization of glioma cellular heterogeneity (3). Single-cell analysis is proving informative about cell subpopulations in normal tissues and in treated recurrent GBM, with the latter providing insights regarding therapy-driven tumor evolution (4, 5). It is anticipated that single-cell analyses will ultimately prove informative regarding the individualization of glioma patient treatment based on knowledge of a tumor’s cellular heterogeneity combined with increased understanding of cell subpopulation interactions. The myeloid compartment is the predominant subset of immune cells within the GBM microenvironment (6). This myeloid-rich environment is a hallmark of GBM, and these cells exert pro- and anti-tumor influence under different circumstances (6–8). Advances in understanding of glioma cellular heterogeneity from single cell analyses have exemplified the over-simplistic nature of the historically proposed pro-inflammatory M1 and immune suppressive M2 categories. Changes to the M1/M2 classification have been proposed by others, but without the benefit of single-cell characterization data (9). In this review, we discuss the current myeloid classification and its shortcomings, as well as how emerging single-cell technologies can be leveraged for increased understanding of glioma-infiltrating myeloid cell function in addition to impacting clinical outcomes in glioma patients.

### Origins of M1/M2 Macrophage Classification

It was widely accepted for many years that the origin of tissue macrophages could be traced solely to circulating blood monocytes, which would travel to the destination tissue and differentiate into tissue-specific macrophages (i.e. microglia in the central nervous system (CNS), alveolar macrophages in the lungs, Kuppfer cells in the liver, etc.) (10–12). The current understanding is that there is a subset of tissue macrophages such as microglia derived not from circulating monocytes, but rather from stem cell populations found in the yolk sac and fetal liver during embryonic development that endure throughout life, independent of the circulating monocyte population (13–17). Evidence for this includes that these yolk sac-derived macrophages do not rely on the transcription factor c-Myb, which is necessary for differentiation of erythroid-myeloid progenitors into monocytes prior to differentiation into macrophages (18), providing a clear distinction from monocyte-derived macrophages (19, 20).

The M1/M2 classification was originally proposed to subclassify macrophages on the basis of immune activation and functional role, with M1 referring to those that are classically-activated and M2 referencing those that are alternatively activated (21). As shown by Mills et al. specifically in the context of differentiation of bone marrow-derived myeloid cells, macrophages are activated in two different ways, yielding two distinct phenotypes that have antagonistic effects on inflammation (22). Classical activation *via* stimulation with interferon (IFN)-γ, lipopolysaccharide (LPS), or granulocyte-macrophage colony-stimulating factor (GM-CSF), results in an antitumor phenotype in which numerous pro-inflammatory cytokines are produced. Alternative macrophage activation *via* interleukin (IL)-4, IL-10, IL-13, transforming growth factor (TGF)-β, and colony-stimulating factor (CSF)-1 results in the tumor-supportive phenotype characterized by macrophage production of high amounts of anti-inflammatory cytokines such as IL-10 and TGF-β (23). Tumor-supportive glioma-associated macrophages (GAMs) cells suppress inflammation, impairing the anti-tumor activity of effector cells such as T cells and natural killer (NK) cells, in addition to inducing other immunosuppressive cells such as Treg cells that ultimately support tumor growth and metastasis. A higher ratio of tumor-supportive GAMs to antitumoral GAMs is associated with a worse prognosis for cancer patients (24).

## Discussion

### Complex Heterogeneity of Tumor-Associated Macrophages in GBM

A number of immune cell populations have been identified throughout the glioma TME, specifically macrophages, resident microglia, T and B lymphocytes, NK cells, and neutrophils, implying that the CNS is far from immune-privileged as was once thought to be the case (25–29). In fact, recent estimates suggest that 30-50% of tumor tissue is composed of monocyte-derived macrophages and microglia, which are the most numerous immune cell populations in GBM (30). The phenotypic profile of the TME immune population is subject to multiple factors dependent not only on the glioma type, but also on the location within the TME. Tumor mutational status appears to have significant impact on TME macrophage state and phenotype as well. In a comparison of isocitrate dehydrogenase (*IDH*) wild-type to *IDH* mutant gliomas, one study found that midkine (a neuroinflammatory cytokine that promotes macrophage polarization to an M2 phenotype) was preferentially upregulated in CD45+ myeloid cells of *IDH* wild-type gliomas as compared to *IDH* mutated gliomas (6). Additionally, GAMs in *IDH* wild-type tumors have been found to express higher levels of anti-inflammatory annexin A1 (ANXA1) and glycoprotein NMB (GPNMB) that have previously been found to be pro-tumorigenic (6). Regarding immune cell composition, GAMs are the most abundant in *IDH* wild-type tumors, while microglia were more common in *IDH* mutant tumors (6). These findings further highlight the need for a more granular investigation into the complex immune dynamics at play within the glioma TME.

TCGA research has revealed three molecular classifications for GBM: classical, proneural, and mesenchymal - each with distinct expression patterns that influence local macrophage polarization and gene expression (3, 25, 31, 32). Tumors of the mesenchymal subtype exhibit the highest expression of immunosuppressive genes that transcribe for galectin-3, IL-10, IL-23, and TGF-β and pro-inflammatory genes that transcribe for IL-2 and IFN (33). Conversely, recent evidence suggests that macrophages influence the phenotype of GBM cells to a mesenchymal-like state that involves the upregulation of MHC class I and II (34). This preferential expression of pro- and anti-inflammatory genes not only promotes macrophage polarization within the TME but may also render the mesenchymal subtype of GBM more amenable to immunotherapeutic approaches. Prospective evaluation of this hypothesis requires GBM molecular classification in clinical trials that test the efficacy of immunotherapeutic treatments.

Recent work using single-cell sequencing techniques has shown significant insight into the phenotypic heterogeneity of macrophages within the glioma TME. scRNA-seq of GBM and low-grade gliomas (LGGs) has revealed that TAMs co-express canonical markers associated with antitumor and tumor-supportive macrophage polarization, with 66% of examined GAMs expressing both the immunosuppressive marker IL-10 and the pro-inflammatory marker TNF-α (35, 36). These results were consistent with the analytical techniques used, with flow cytometry revealing co-expression of immune costimulatory marker CD86 and the immunosuppressive marker CD206. The findings of macrophage co-expression of heterogeneous pro- and anti-tumor markers are corroborated by the results in a number of other studies (37–39). It is worth mentioning here that the expression of such markers can rapidly change in association with treatment, as indicated by the results of studies in which GBM patients received co-treatment with rapamycin and hydroxychloroquine or concurrent stereotactic radiotherapy with immune checkpoint blockade *via* programmed cell death protein 1 (PD-1) signal disruption (40, 41).

Recent studies have revealed transcriptional variability among monocyte-derived macrophages and microglia within the TME of glioma and brain metastases, with expression profiles not fitting into the classic M1 versus M2 polarization paradigm. One study found that high levels of traditionally “M1 markers” such as IL-6 and IL-1β were expressed by the same macrophages expressing traditionally “M2 markers” like matrix metalloproteinase (MMP)-1 and fibronectin 1 (FN1) (6). It has been shown that CD45+ myeloid cells in the GBM TME express markers of immune activation as well as immune suppression (42). A preclinical study detailed that treatment with anti-PD-1 therapy induced macrophage and microglia polarization towards a proinflammatory phenotype in glioma of CD8^-/-^ mice, suggesting that the therapeutic effect of PD-1 blockade may be due to innate rather than adaptive immune system function (28). These findings underscore the complex plasticity of glioma cells to phenotypically adapt to different environments reflected in distinct transcriptional and evolutionary patterns for each patient. Nonetheless, this variability is subject to subclassification categories that may have implications for patient-specific treatments (3, 43–45).

Combined transcriptomic and proteomic approaches such as CITE-seq have demonstrated the ability to define the multidimensionality of myeloid cells and to delineate the spectrum of functions that these immune cells can display in the context of gliomas (46, 47). For instance, in applying single-cell sequencing to GAMs from human GBMs, Pombo Antunes proposed five distinct subtypes based on the activation state of the macrophage: transitory (showing markers of both monocyte and macrophage genes); phagocytic with lipid metabolism; hypoxic and glycolytic; *SEPP1* ^low^, and *SEPP1* ^high^ (46). Notably, this diversity of macrophage states was recapitulated in murine gliomas analyzed with the same approach involving simultaneous proteomic and transcriptomic characterization. While this is but one way to further organize macrophage states, it may provide more utility than the existing classifications.

Another example of mouse and human data integration is an scRNA-seq study that delineated differences in the transcriptional networks between microglia and macrophages derived from non-tumor bearing mice and those derived from glioma-bearing mice. In this study, the expression of genes encoding the MHC class II molecule were increased in GAMs compared to myeloid cells isolated from non-tumor bearing mice. This difference in expression of MHC class II-associated genes was further appreciated in activated microglia from male tumor-bearing mice and GBM patients (48). Although these data show the potential antigen presentation capabilities of GAMs, scRNA-seq analysis of murine and human gliomas has also shown the immunosuppressive nature of these myeloid cells. For instance, one study showed that *ARG1/2* was upregulated predominantly in glioma-associated macrophages as opposed to microglia (49). This was further corroborated by elevated arginase-1 levels synthesized by tumor-infiltrating macrophages from mouse and human gliomas that promote the generation of polyamines and thus, T cell suppression (50).

In sum, these studies show the resolution that emerging single-cell technologies possess to characterize different transcriptional states of glioma-associated microglia and macrophages allowing the conceptualization of their complexity and heterogeneity across species.

### Deconvolutional Techniques for Immune Characterization in GBM

Advanced single-cell analysis techniques like scRNA-seq and CyTOF provide an unprecedented level of resolution in characterizing the cellular composition of the TME. However, there remain several limitations with these techniques, particularly as they pertain to glioma research. CyTOF requires the selection of cell-surface markers, and although the number of detectable surface markers is rapidly growing, there is still a limitation to the absolute number that can be analyzed at one time, such that informative marker combinations can be missed due to initial marker selection. In addition, there is no standard technique for the processing of CyTOF data, leading to differences in results between labs that have performed CyTOF using the same set of markers. In particular, the randomization transformation used to better visualize CyTOF results is inherently different across analyses, and thus it has been suggested that raw data and detailed methods be provided for subsequent analysis whenever conclusions are reached from CyTOF data (51).

New results from scRNA-seq, on the other hand, are more easily compared against existing results given the vast amount of accessible online data (GBMseq, Ivy GAP, TCGA). For example, several novel techniques have been developed recently using scRNA-seq to investigate cellular interactions and resulting transcriptomes on a single cell level in the TME such as RABID-seq and PIC-SEQ (52–54). However, considering that the transcription of a portion of the genome occurs as episodic and pulsatile bursts and that RNA collection and analysis is a snapshot in time, differential clusters of RNA expression seen during one analysis can be drastically different at another point in time (55). Similarly, phenotypic states of the same cell type as well as the immune cell composition within a TME changes with time and tumor evolution (55). Another limitation inherent to RNA expression analysis is that RNA expression does not explicitly translate to protein expression, resulting in only prediction of the potential cellular activities that might be occurring in the TME. Therefore, transcriptomics should be complemented with proteomics, functional assays, and spatial analysis. Furthermore, as a deconstructive and disruptive technique, the spatial relationships involving cell-cell interactions that influence transcriptional states are lost during the process of cell isolation, and accordingly are extrapolated from imaging data, which, even if subject of a certain degree of accuracy, is still at risk of error.

The spatial evaluation at the single-cell level along with functional information is the next step in characterizing the TME in finer detail. These will allow for more extensive investigations into the genetic and cellular changes that occur in response to therapeutic intervention within the TME, where within the TME these changes are occurring, and how best to exploit them to improve clinical response and outcomes. Previous investigations have examined the spatial distribution of immune cells throughout the infiltrating edge, proper tumor, and necrotic core of glioma, and found that there is significant heterogeneity throughout these areas in immune cell composition, distribution, and interactions (43). However, these spatial relationships between immune cells are only just starting to be further investigated in GBM undergoing microenvironmental change in response to therapy, allowing for unique opportunities for single-cell techniques to provide novel insight with the potential to advance therapeutic efficacy.

All shortcomings considered with respect to the different types of single cell analysis, the best approach for maximizing informative and accurate information yield is to utilize multiple techniques combined with spatial mapping of sample acquisition. Methods to integrate genomics, transcriptomics, and spatial measurements are emerging and have increasing influence on the way tumors are studied. Recently, Zhao et al. described a promising spatial genomic technique that not only allows for the detection of different clones of cells that harbor distinct genomic signatures, but also correlates signatures with cell location within the TME. For this particular study, spatial genomic heterogeneity was focused on tumor cells, but the approach can certainly be applied to other TME cell types such as GAMs (56). With the continuing refinement of spatial multiplex imaging technologies, detailed characterizations of the immune proteome within the TME have become possible. Using these techniques, one study found that myeloid cells localized to mesenchymal-like regions of GBM drive T cell exhaustion *via* IL-10 release, and that this T cell function was rescued with Janus kinase-signal transducer and activator of transcription (JAK/STAT) inhibition, providing a potential therapeutic opportunity (57). These techniques are allowing for the discovery of wider and more heterogeneous populations of immune cells with newly identified, previously unexploited cell phenotypes with distinct functions that influence tumor biology.

### Leveraging Data From Single-Cell Technologies in Gliomas

**Figure 1:**
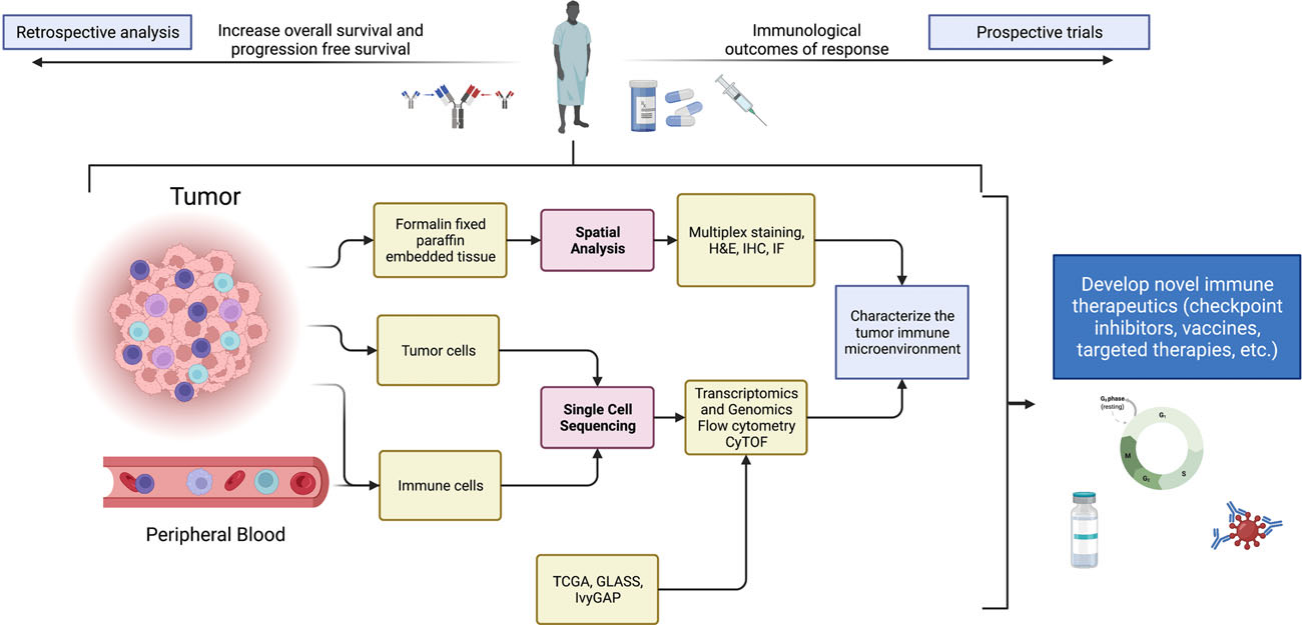
Summary schematic detailing sources of samples, analytical techniques, and potential uses of data from single-cell technologies to improve and devise novel immune therapeutics. Specifically, single-cell technologies can be implemented in clinical studies for biomarker discovery, characterization of novel drug targets, and the study of pharmacodynamic effects of therapies in window-of-opportunity clinical trials. Created with BioRender.

Single cell technologies are providing opportunities to leverage data for clinical trial design and especially for treatment response interpretation (**Figure 1**). In instances where biopsy sampling of tumor prior to the initiation of treatment is possible, cellular profiles of pre-treatment specimens and corresponding on-therapy specimens obtained during surgical resection can be compared for determining the effect of treatment on TME cellular composition, as well as for determining the presence or absence of treatment anti-tumor activity. Single-cell analyses can also be used in a retrospective manner to study patient outcomes. One can conduct a retrospective analysis of tissue from patients that have received a common treatment, and in instances where tumor is collected post-mortem, the data obtained from end-stage tumors would prove informative regarding tumor evolution in response to a specific therapy. Importantly, mass cytometry and multiplex immunofluorescence can be used when specimen availability is limited to fixed tissues. In instances where frozen tissue is available, there are protocols for isolating nuclei that, in turn, enables single-nuclei RNA-seq (58). An example of this type of retrospective strategy that used fixed tissues found an association of extracellular signal-regulated kinases (ERK) 1/2 phosphorylation, an indicator of mitogen activated protein kinase (MAPK) pathway activation, with increased response and survival to adjuvant anti-PD-1 therapy in independent cohorts of recurrent GBMs (59). The integration of single-cell transcriptome analysis and multiplex immunofluorescence showed that tumors with an abundance of p-ERK contained GAMs with high major histocompatibility complex (MHC) class II gene and protein expression.

A final application of single cell analyses concerns immunoediting whereby GBM cells acquire an immune escape phenotype. Following treatment with standard-of-care temozolomide (TMZ) and radiotherapy (RT), changes in macrophage differentiation and polarization, as well as alterations in T-lymphocyte populations have been observed (60). Further, scRNA-seq data was used to investigate changes in GAM populations in response to radiotherapy specifically, finding that there was an increased ratio of macrophage:microglia as well as increased alternative activation in both populations leading to a predominantly immunosuppressive phenotype. The same study found that blocking these radiation-induced changes *via* administration of CSF-1R inhibitors significantly increased survival in preclinical models (61). Another study showed that tumors treated with neoadjuvant PD-1 immune checkpoint blockade contained higher numbers of CD3^+^ T cells, with further analysis of the T cell receptor repertoire showing that signatures from peripheral and tumor-infiltrating T cells overlapped, suggesting that T cells from the circulation infiltrated these gliomas (62). Importantly, PD-1 blockade induced an IFN-γ gene signature in glioma-infiltrating monocytes and macrophages that was reflected by the expression of *CXCL9/10* and *PD-L1*. While the use of single-cell technologies has not yet become routine in patient care, these findings all suggest multiple applications of such single-cell techniques to provide novel avenues for therapeutic intervention.

## Conclusion

The complexity of intratumor heterogeneity represented by diverse gene expression programs of cancer and immune cell populations in the TME has yet to be exploited for the benefit of patients. The analysis of tumor and immune cells, using single-cell technologies such as scRNA-seq and CyTOF, either before or after therapy, has the ability to dissect in detail the mechanisms of therapeutic response and resistance. In the future, we expect that integrative approaches involving the application of these methods will provide data that advance personalized treatments for cancer patients, and that will lead to improved treatment outcomes.

## Author Contributions

All authors have contributed significantly towards this manuscript as follows: (1) Conception and Design: AH, VA, HN, and CL; (2) Administrative Support: CL; (3) Provision of Study Materials or Patients: N/A; (4) Collection and Assembly of Data/Information: CL, VA, and HN; (5) Data Analysis and Interpretation: All authors; (6) Manuscript Writing: All authors; (7) Final Approval of Manuscript: All authors.

## Funding

Research support was provided by the Northwestern Medicine Malnati Brain Tumor Institute of the Lurie Cancer Center, and NIH grants NS120547, CA120813, and CA221747.

## Conflict of Interest

The authors declare that the research was conducted in the absence of any commercial or financial relationships that could be construed as a potential conflict of interest.

## Publisher’s Note

All claims expressed in this article are solely those of the authors and do not necessarily represent those of their affiliated organizations, or those of the publisher, the editors and the reviewers. Any product that may be evaluated in this article, or claim that may be made by its manufacturer, is not guaranteed or endorsed by the publisher.
